# The negative association between pre-eclampsia and breast cancer risk may depend on the offspring's gender

**DOI:** 10.1038/sj.bjc.6603688

**Published:** 2007-03-27

**Authors:** L J Vatten, M R Forman, T I L Nilsen, J C Barrett, P R Romundstad

**Affiliations:** 1 Department of Public Health, Faculty of Medicine, Norwegian University of Science and Technology, Trondheim, Norway; 2 MD Anderson Cancer Center, Houston, TX, USA; 3 Laboratory of Biosystems and Cancer, National Cancer Institute, Bethesda, MD, USA

**Keywords:** pre-eclampsia, offspring's gender, breast cancer, epidemiology

## Abstract

If the negative association between pre-eclampsia and subsequent breast cancer risk differs by gender, this would strengthen the hypothesis that factors intrinsic to the particular pregnancy may explain the association. The study included 701 006 parous Norwegian women with follow-up for breast cancer through the Cancer Registry of Norway. Breast cancer risk was lower in women with pre-eclampsia/hypertension in their first pregnancy, compared to other women (relative risk, 0.86, 95% CI, 0.78–0.94), after adjustment for age at first birth, maternal birth year, length of gestation, marital status, and parity. The risk reduction was slightly greater if the woman delivered a son as opposed to a daughter (relative risks of 0.79 *vs* 0.94, *P*-value for interaction, 0.06), and if pre-eclampsia/hypertension was combined with pre-term delivery, these differences were more pronounced (relative risks, 0.62 *vs* 1.07, *P*-value for interaction 0.03). A subanalysis among 176 036 primiparous women showed a substantial risk reduction if the mother delivered a son (relative risk, 0.62, 95% CI, 0.47–0.82), but essentially null if she delivered a daughter (relative risk, 0.92, 95% CI, 0.72–1.18; *P*-value for interaction, 0.05). These results suggest that the effect of pre-eclampsia/hypertension may be attributed to factors associated with the particular pregnancy rather than an underlying biological trait of the mother. The stronger risk reduction related to having a son suggests a role for sex-dependent hormones in pregnancy.

Women who are diagnosed with pre-eclampsia in pregnancy may have a lower risk for breast cancer later in life compared to other parous women (Polednak and Janerich, 1983; Thompson *et al*, 1989; Troisi *et al*, 1998; Cohn *et al*, 2001; Vatten *et al*, 2002a, 2002b) although a higher risk has also been reported (Paltiel *et al*, 2004). Women who develop pre-eclampsia tend to have higher levels of androgens and lower oestrogen and progesterone levels than women in normotensive pregnancies (Innes and Byers, 1999). These findings have been linked to a deficiency in aromatisation of androgens in the placenta, which are suggested to be of importance for the reduced risk of breast cancer associated with pre-eclampsia (Troisi *et al*, 1998; Hoover and Troisi, 2001).

The influence of pre-eclampsia on subsequent breast cancer risk could be attributed to factors associated with the particular pregnancy, but the effect could also reflect an underlying biological trait in a woman that decreases the risk for developing breast cancer. One approach that may distinguish between these possibilities would be to study whether the association with pre-eclampsia is modified by pregnancy-specific factors, for example offspring gender. If breast cancer risk associated with pre-eclampsia differs according to gender of the offspring the hypothesis that the effect of pre-eclampsia could originate from that particular pregnancy would be strengthened.

In a cohort of 701 006 women in Norway, pregnancy-related information from the Medical Birth Registry was linked to the National Cancer Registry. The aim of the study was to compare breast cancer risk in women who had experienced pre-eclampsia or hypertension in their first pregnancy to the risk of women who were normotensive, and to study whether gender of the offspring modifies the association.

## MATERIALS AND METHODS

Data were derived from the Medical Birth Registry of Norway that comprises all births since 1967, and the Norwegian Cancer Registry, which has registered incident cancers since 1953. Midwives and doctors complete a standardised form to notify the Medical Birth Registry about each birth in the country (www.mbr.no). Pre-eclampsia and gestational hypertension were recorded as indicated in the standardised form of the Medical Birth Registry and described elsewhere (Lie *et al*, 1998). The reporting of cancer to the Cancer Registry by doctors or hospital departments is mandatory and regulated by law (www.kreftregisteret.no). Using the unique identification number of Norwegian citizens, we linked all women registered at the Medical Birth Registry to the national Cancer Registry, and identified women who had developed cancer after delivery. A total of 751 581 women were recorded with a singleton first birth in the Medical Birth Registry between 1967 and 1998. Women with missing or improbable combinations of the offspring's sex, gestational age or birth weight in their first delivery were excluded from the analyses (*n*=48 030). We also excluded women who delivered before 22 weeks of gestation or whose offspring's birth weight was less than 500 g (*n*=880). Women diagnosed with cancer before the beginning of follow-up (*n*=1345), and women lost to follow-up (*n*=320) were also excluded, leaving 701 006 women for analysis. For these women information was available on length of gestation, birth weight, and the offspring's gender. By linkage to the Cancer Registry, we followed these women from the time of delivery (1967–1998) until the diagnosis of cancer, until death from any cause, or to the end of follow-up (31 December, 2002), whichever occurred first.

Most cases of pre-eclampsia occur in the first pregnancy, and including subsequent pregnancies in the analysis might introduce methodological problems that may interfere with the aim of the study. In a subgroup analysis, we therefore analysed the data separately for the 176 036 women who remained primiparous throughout the study period.

We calculated hazard ratios as estimates of the relative risk for breast cancer among women who had experienced pre-eclampsia or hypertension in the index pregnancy compared to the risk of normotensive women. Further, we stratified the analysis by the offspring's gender, to study whether the association between pre-eclampsia and breast cancer risk differed between women who delivered a son or a daughter. In some analyses, we also stratified pre-eclampsia according to pre-term (<37 weeks of gestation) or term (⩾37 weeks) delivery. In the analysis, we adjusted for potential confounding by the women's year of birth (5 year categories), age at delivery (5 year categories), length of gestation (completed weeks), parity, and marital status, using Cox regression analysis (Stata Statistical Software: Release 9, College Station, TX, USA). Precision of the relative risks was estimated by 95% confidence intervals, and the interaction between pre-eclampsia and offspring gender was statistically tested in the regression model.

## RESULTS

During follow-up, a total of 9160 incident cases of breast cancer were diagnosed among 701 006 women (Table 1). Among cases, 503 were diagnosed among 43 286 women with pre-eclampsia or hypertension in pregnancy. Compared to normotensive women, the risk of breast cancer was lower among women who had pre-eclampsia or gestational hypertension in their first pregnancy (relative risk 0.86, 95% CI, 0.78–0.94), after adjustment for maternal birth year, age at delivery, length of gestation, parity, and marital status.

In women who delivered a son, the risk reduction was slightly greater (relative risk 0.79, 95% CI, 0.69–0.90) than for women who had a daughter (relative risk 0.94, 95% CI, 0.82–1.06), but the test for interaction was of borderline significance (*P*=0.06). If pre-eclampsia/hypertension was combined with pre-term delivery, the risk reduction associated with having a son and not a daughter was even greater (relative risks, 0.62 *vs* 1.07, *P*-value for interaction 0.03).

In a subgroup of 176 036 women who remained primiparous throughout the study period, a total of 1916 incident cases of breast cancer were diagnosed during follow-up, and 121 of these cases occurred among the 11 945 women who had pre-eclampsia or hypertension in pregnancy (Table 2). Similar to the finding for the whole population the risk of breast cancer was lower among women who had pre-eclampsia or gestational hypertension (relative risk 0.75, 95% CI, 0.62–0.91), after adjustment for age, age at delivery, length of gestation, and marital status. Also in primiparous women, those who delivered a son had a stronger reduction in breast cancer risk (relative risk 0.62, 95% CI, 0.47–0.82) compared to women who delivered a daughter (relative risk 0.92, 95% CI, 0.72–1.18; *P*-value for interaction 0.05). Further stratification by gestational age (pre-term or term pre-eclampsia) among the primiparous women was not calculated because of the small number of breast cancers in women with pre-term pre-eclampsia.

## DISCUSSION

We found that the reduced risk of breast cancer associated with pre-eclampsia or hypertension in first pregnancy was substantial among women who had a son, but essentially null in women who delivered a daughter. Previous studies have also shown an inverse association related to pre-eclampsia (Polednak and Janerich, 1983; Thompson *et al*, 1989; Troisi *et al*, 1998; Cohn *et al*, 2001; Vatten *et al*, 2002a, 2002b), but no previous study has assessed whether the offspring's gender could modify this association.

Clinical information included in health registries is rarely validated, partly because a gold standard for the diagnosis is not available. Although this may be a weakness of this study, it is equally clear that the diagnosis of pre-eclampsia (or gestational hypertension) in the Medical Birth Registry did not depend on future diagnosis of breast cancer. Consequently, any bias in the association between pre-eclampsia or gestational hypertension and subsequent breast cancer risk is likely to be non-differential, and lead to underestimates of the real effect.

In the general population, shorter lengths of gestation appear to reduce the pregnancy-related protection against breast cancer (Vatten *et al*, 2002a, 2002b), indicating that mechanisms operating during the third trimester (e.g. the androgen surge at term) are particularly important for protection. Pre-eclampsia in its severe form often results in early induction of delivery so it is appropriate to account for length of gestation. In our study, however, adjustment for gestational length did not appreciably change the results.

An inverse association between pre-eclampsia and breast cancer risk has now been confirmed in a number of studies. It is, however, not clear if the reduction in risk is attributable to long-term effects of the particular pregnancy, or whether women who develop pre-eclampsia exhibit an underlying biological trait that is somehow protective against breast cancer development.

A combination of genetic susceptibility and abnormal placentation is thought to constitute the basis for pre-eclampsia, but clinically, the severity of the syndrome depends on degree of hypertension and proteinuria from 20 weeks of gestation and onwards. Pre-eclampsia is characterised by a disturbance in the fundamental balance between steroid and other pregnancy hormones during pregnancy (Roberts and Cooper, 2001). Maternal androgen levels appear to be higher than in normotensive pregnancies, and levels of oestrogen and progesterone appear to be lower. Thus, it has been suggested that the reduced risk of breast cancer associated with pre-eclampsia could be attributed to lower concentrations of oestrogen and progesterone (Innes and Byers, 1999). It has also been suggested that an androgen influence associated with the pre-eclamptic condition may be important (Hoover and Troisi, 2001). Future research needs to address the cyclical fluctuation of hormones in women who have had a diagnosis of pre-eclampsia compared to normotensive women. This research would best be conducted in primiparous women to reduce hormonal influences of subsequent pregnancies.

Our results suggest that the protective effect associated with pre-eclampsia was restricted to women who delivered a son. This finding may support the hypothesis that a protective effect of pre-eclampsia on breast cancer risk could originate from the particular pregnancy, rather than indicating an underlying biological trait that is protective against breast cancer in women who develop pre-eclampsia.

## Figures and Tables

**Table 1:** Relative risk of breast cancer, comparing women who had pre-eclampsia or gestational hypertension in their first pregnancy, and women who were normotensive, by gender of the offspring

**Characteristics of pregnancy**	**No. of women**	**Breast cancer**	**Relative risk**	**(95% CI)**
*All pregnancies*				
Normotensive	657 720	8657	1.0	(Reference)
Pre-eclampsia or hypertension	43 286	503	0.86^a^	(0.78–0.94)
Pre-term^c^ pre-eclampsia/ hypertension	5482	60	0.84^a^	(0.64–1.09)

*Male offspring*				
Normotensive	338 129	4468	1.0	(Reference)
Pre-eclampsia or hypertension	22 984	246	0.79^b^	(0.60–0.90)
Pre-term^c^ pre-eclampsia/Hypertension	2956	24	0.62^b^	(0.42–0.95)

*Female offspring*				
Normotensive	319 591	4194	1.0	(Reference)
Pre-eclampsia or hypertension	20 302	252	0.94^b^	(0.82–1.06)
Pre-term^c^ preeclampsia/hypertension	2526	36	1.07^b^	(0.75–1.52)

a Adjusted for maternal age, age at first birth, length of gestation, parity, marital status, and offspring gender.

b Adjusted for maternal age, age at first birth, length of gestation, parity, and marital status.

c Pre-term: delivery before 37 weeks of gestation.

CI = confidence interval.

Significance test for interaction between pre-eclampsia and offspring gender, *P*=0.06.

Significance test for interaction between pre-term pre-eclampsia/hypertension and offspring gender, *P*=0.03.

**Table 2:** Relative risk of breast cancer, comparing primiparous women who had pre-eclampsia or gestational hypertension in pregnancy, and primiparous women who were normotensive, by gender of the offspring

**Characteristics of pregnancy**	**Primiparous women**	**Breast cancer**	**Relative risk**	**(95% CI)**
*All pregnancies*				
Normotensive	164 091	1795	1.0	(Reference)
Pre-eclampsia or hypertension	11 945	121	0.75^a^	(0.62 to 0.91)

*Male offspring*				
Normotensive	84 274	908	1.0	(Reference)
Pre-eclampsia or hypertension	6199	51	0.62^b^	(0.47–0.82)

*Female offspring*				
Normotensive	79 817	887	1.0	(Reference)
Pre-eclampsia or hypertension	5746	70	0.92^b^	(0.72–1.18)

a Adjusted for maternal age, age at birth, marital status, and offspring gender.

b Adjusted for maternal age, age at birth, and marital status.

CI = confidence interval.

Significance test for interaction between pre-eclampsia and offspring gender, *P*=0.05.
